# Mechanisms of Therapeutic Resistance in Cancer (Stem) Cells with Emphasis on Thyroid Cancer Cells

**DOI:** 10.3389/fendo.2014.00037

**Published:** 2014-03-25

**Authors:** Sabine Hombach-Klonisch, Suchitra Natarajan, Thatchawan Thanasupawat, Manoj Medapati, Alok Pathak, Saeid Ghavami, Thomas Klonisch

**Affiliations:** ^1^Department of Human Anatomy and Cell Science, University of Manitoba, Winnipeg, MB, Canada; ^2^Department of Obstetrics, Gynecology and Reproductive Sciences, University of Manitoba, Winnipeg, MB, Canada; ^3^Department of Surgery, University of Manitoba, Winnipeg, MB, Canada; ^4^Manitoba Institute of Child Health, University of Manitoba, Winnipeg, MB, Canada; ^5^Department of Medical Microbiology and Infectious Diseases, University of Manitoba, Winnipeg, MB, Canada

**Keywords:** thyroid cancer, therapeutic resistance, stem cells, HMGA2, DAMP, autophagy, ER stress, DNA repair

## Abstract

The two main reasons for death of cancer patients, tumor recurrence and metastasis, are multi-stage cellular processes that involve increased cell plasticity and coincide with elevated resistance to anti-cancer treatments. Epithelial-to-mesenchymal transition (EMT) is a key contributor to metastasis in many cancer types, including thyroid cancer and is known to confer stem cell-like properties onto cancer cells. This review provides an overview of molecular mechanisms and factors known to contribute to cancer cell plasticity and capable of enhancing cancer cell resistance to radio- and chemotherapy. We elucidate the role of DNA repair mechanisms in contributing to therapeutic resistance, with a special emphasis on thyroid cancer. Next, we explore the emerging roles of autophagy and damage-associated molecular pattern responses in EMT and chemoresistance in tumor cells. Finally, we demonstrate how cancer cells, including thyroid cancer cells, can highjack the oncofetal nucleoprotein high-mobility group A2 to gain increased transformative cell plasticity, prevent apoptosis, and enhance metastasis of chemoresistant tumor cells.

## Introduction

Tissue invasion, metastasis, as well as radio- and chemotherapeutic resistance to anti-cancer treatments are common and main causes of death in cancer patients. Tumor cells mount complex and still poorly understood molecular defense mechanisms to counteract and evade oxygen deprivation, nutritional restrictions, as well as radio- and chemotherapeutic treatment regimens aimed at destabilizing their genomes and important cellular processes. In thyroid cancer, as in other tumors, such defense strategies include the reactivation in cancer cells of early developmental programs normally active exclusively in stem cells, the stimulation of cancer stem-like cells resident within the tumor tissue, and the recruitment of bone marrow-derived progenitors into the tumor (1–3). Metastasis and therapeutic resistance in cancer (stem) cells involve the epithelial-to-mesenchymal transition (EMT)-mediated enhancement in cellular plasticity, which includes coordinated dynamic biochemical and nuclear changes (4). The purpose of the present review is to provide an overview of the role of DNA repair mechanisms contributing to radio- and chemotherapeutic resistance in cancer with an emphasis on thyroid cancer and highlight the emerging roles of autophagy and damage-associated molecular pattern (DAMP) responses in EMT and chemoresistance in tumor cells. Finally, we use the stem cell factor and nucleoprotein high-mobility group A2 (HMGA2) as an example to demonstrate how factors intended to protect stem cells are wielded by cancer (stem) cells to gain increased transformative cell plasticity, which enhances metastasis, chemotherapeutic resistance, and cell survival. Wherever possible, we have included information on these cellular processes and associated factors as they relate to thyroid cancer cells.

## Thyroid Cancer: High Incidence and New Ways to Predict Risk of Death

Thyroid cancer is the most common malignant endocrine tumor and the seventh most common cancer seen in Canadians accounting for 11% of all cancers in women <40 years. In Canada, the incidence of thyroid cancer is increasing more rapidly than any other cancer; by 6.8% per year in Canadian males (1998–2007) and by 6.9% per year in Canadian females (2002–2007) (5). A 373% increase in the incidence of thyroid cancer was reported in a population-based cohort in Canada (6). The trends in the United States (US) mirror that of Canada, with an increase in the incidence of thyroid cancer from 4.85/100,000 in 1975 to 14.25/100,000 in 2009 and an annual percent increase (2000–2009) of 6.0% for the US males and 6.9% for the US females (7). The life time probability of developing a thyroid cancer for a Canadian female is 1 in 71 (1.4%) but only 1 in 1,374 (0.1%) will actually die from it. Canadian males have a lower lifetime risk of developing thyroid cancer at 1 in 223 (0.4%) with the risk of death from thyroid cancer at 1 in 1,937 (0.1%) (8). Although the incidence of thyroid cancer has been rising, this tumor has an excellent 5-year relative survival ratio of 98% in 2011 (8). Thyroid cancer represents a conglomerate of different histological types with diverse clinical behavior. Over 90% of all thyroid cancers are either follicular or papillary carcinoma, termed differentiated thyroid cancer (DTC), and carry excellent prognosis. By contrast, poorly differentiated and anaplastic thyroid cancers (ATC) have a very poor outcome. Surgery and/or radioactive iodine exposure is the mainstay of treatment for DTC. ATC are usually diagnosed at an advanced stage when surgery is not feasible and radiation and chemotherapy are the only option. Thyroid cancer stem cell populations have been described for both DTC and ATC (2, 9–11). The histology and age of the patient at diagnosis are two principal determinants of thyroid cancer-specific survival. The improvement in the thyroid cancer-specific survival over the last four decades is largely attributed to the declining proportion of ATC (12). An age threshold of 45 years at the time of diagnosis of DTC used by the TNM classification system of the American Joint Committee on Cancer and International Union against Cancer (TNM-AJCC/IUCC) for stratification into low- and high-risk thyroid cancers has been questioned and an alternative age cut off of 55 years was suggested (13). To predict an individual’s risk of death from thyroid cancer within 10 years of diagnosis, we recently developed a prognostic nomogram which accounts for the patient’s age and gender, TNM stage and histology, and the presence of post-treatment macroscopic residual disease as important independent determinants of thyroid cancer-specific survival (12).

## Mechanisms of Therapy Resistance in Thyroid Cancer

### DNA repair mechanisms

Mechanisms involved in single-stranded [base excision repair (BER), nucleotide excision repair (NER), mismatch repair (MMR)] and double-stranded [homologous recombination (HR), non-homologous end-joining (NHEJ)] DNA repair significantly affect the ability of thyroid cancer (stem) cells to counteract and survive radio- and chemotherapy. Here, we focus on factors and their polymorphic genotypes that are involved in specific DNA repair pathways and have been shown to affect the incidence of thyroid cancer.

The multistep BER pathway is the main mechanism responsible for the replacement of individual DNA bases that have been altered by alkylation, oxidation, and deamination [for review see Ref. (14)]. The damaged base is recognized by specific DNA glycosylases like OGG1, which recognizes and excises 8-oxoguanine to generate an apurinic/apyrimidinic (AP) abasic site. Loss of heterozygosity (LOH) of the glycosylase OGG1 is strongly associated with papillary thyroid cancer (PTC) (15). The apurinic/apyrimidinic endonuclease 1 (APE1) recognizes the AP site and cleaves the phosphodiester bond at the 5′ end to yield a 3′-OH nucleotide and 5′-deoxyribose phosphate (dRp) terminus. The 5′-dRp site is cytotoxic and conversion into an inert 3′-OH requires lyase activity provided by BER-associated APE1, DNA polymerase β (PolB), and stem cell nucleoprotein members high-mobility group A1 and A2 (HMGA1/2) (16). Presence of HMGA1 and/or HMGA2 confers enhanced BER capacity and chemoresistance against alkylating agents onto thyroid cancer cells (16, 17). The scaffolding protein X-ray repair cross-complementing group 1 (XRCC1) facilitates the assembly of the PolB–DNA ligase III–PARP complex and the formation of phosphodiester bonds to complete the BER repair of AP sites (18). Investigations into the possible association of XRCC1 Arg399Gln, Arg280His, and Arg194Trp polymorphisms with thyroid cancer revealed that hetero- and homozygous XRCC1 Arg399Gln polymorphism coincided with a decreased risk of DTC in the Caucasian population and mixed population (19–22). The XRCC1 Arg280His polymorphism enhanced susceptibility of DTC in Caucasians but protect against DTC in Asians (21, 23), whereas a homozygous XRCC1 Arg194Trp polymorphic genotype may increase the risk of PTC and lymphatic metastasis (19, 20, 24, 25).

The ERCC2 DNA helicase is a member of the NER pathway and contributes to the repair of distorted DNA regions due to bulky DNA adducts and UV light-induced DNA damage [for review see Ref. (26)]. The ERCC2 G23591A gene polymorphism results in an Asp312Asn mutation in a conserved region and the A35931C polymorphism causes a Lys751Gln substitution. Both polymorphisms are in linkage equilibrium and those patients homozygous for both rare variant alleles show an increased risk for PTC but not follicular thyroid cancer (FTC) (27).

The MMR pathway eliminates mismatched bases and incorrect insertions/deletions as a result of faulty DNA replication. The heterodimer of MutS complex (MSH2 pairing with MSH6 and MSH3, respectively) locks onto the base mismatch and recruits heterodimeric endonuclease MutL, composed of PMS2 and MLH1, and the exonuclease 1 (EXO1) to remove the mismatched base. The abasic gap is filled and ligated by DNA polymerase δ/ε and DNA ligase, respectively (28). Higher expression of MLH1, MSH2, and PMS1 were observed in malignant thyroid tumors than in benign lesions (29). BRAF V600E mutations, RET/PTC rearrangements, and transitions (IDH1 and NRAS) are associated with low expression of MLH1 in thyroid cancer patients (30).

Genotoxic ionizing radiation and DNA-damaging agents can cause double-strand breaks (DSBs). This triggers DNA DSB repair in the form of either HR or NHEJ (31). HR uses a sister chromatid template and thus functions in late G2–S cell cycle phase. HR is a highly accurate and error-free repair process [for review see Ref. (28, 31, 32)]. Radiation-induced thyroid tumors are the direct result of radiation-related DSBs and chromosomal rearrangements (33–35). In the Saudi Arabian population, the RAD52 Gln221Glu polymorphic genotype and RAD52 2259C > T genotype, and variants thereof carrying a T allele, were reported to have a significantly higher risk of developing thyroid cancer (36). Two other studies identified the combinations of RAD52 2259C > T, XRCC2 R188H and XRCC3 T241M polymorphisms (37) and RAD51 Exon1/59G > T, XRCC3 Thr241Met variant alleles to have predictive value for the polygenic risk of thyroid cancer (19).

Non-homologous end-joining is a DSB repair that does not require homologous DNA as a template and, thus, functions throughout the cell cycle [for review see Ref. (38–40)]. The Ku70/80 protein complex recognizes DSBs and participates in the recruitment of DNA-dependent protein kinases. The human pituitary tumor transforming gene (PTTG) is highly expressed in human thyroid carcinoma and binds to and inhibits Ku70 protein. Suppression of PTTG gene expression coincides with an up-regulation of DNA repair proteins in thyroid cancer suggesting a role for PTTG in therapeutic resistance (41–45). XRCC7 is another NHEJ factor and XRCC7 Ile3434Thr polymorphism has recently been associated with increased incidence of DTC (46).

## Endoplasmic Reticulum Stress, Autophagy, and Epithelial-to-Mesenchymal Transition

### Endoplasmic reticulum stress and epithelial mesenchymal transition

Unfolded protein response (UPR) pathways are activated to offset the adverse effects of protein accumulation in the endoplasmic reticulum (ER), following the induction of ER stress [reviewed in Ref. (47)]. Activating transcription factor 6 (ATF6), inositol-requiring protein-1 (IRE1), and PKR-like ER kinase (PERK) constitute the three arms of the UPR which control specific regulatory mechanisms to ease protein translation, regulate metabolism rate and redox events, augment expression of protein-folding chaperones, and increase the production of protein degradation enzymes (48, 49). The first step of UPR activation includes GRP78 chaperone dissociation from ER membrane-spanning UPR receptor proteins, PERK, IRE1, and ATF6 (UPR arms), to facilitate protein refolding [reviewed in Ref. (49)]. Several metabolic and environmental cues can trigger ER stress induction and initiate UPR pathways. Cancer-related impairment in UPR response might affect the ability of cells to maintain homeostasis during ER stress (50). Rapidly growing tumors are highly dependent on nutrients and oxygen delivered by the tumor vasculature. Activated ER stress stimulates an adaptive UPR process and helps cancer (stem) cells to survive. The X-box binding protein-1 (XBP-1) has an important involvement in UPR-induced cell survival and is induced via the IRE1 pathway of UPR (47, 51). XBP-1 is often over-expressed in cancer cells and, importantly, increases drug resistance by interfering with cell cycle regulation and by down-regulating tumor apoptotic responses to anti-cancer drugs. This makes XBP-1 an attractive cellular factor for dedifferentiation and EMT in breast cancer cells that negatively regulates the organization of polarized epithelial cells in estrogen-dependent and -independent breast tumors (52). Intriguingly, ER stress inducers (thapsigargin and tunicamycin) were shown to increase XBP-1 splicing in PC-Cl3 thyroid cells, impeding the thyroglobulin folding process and inducing accumulation of this glycoprotein in the ER. In the absence of apoptosis, differentiation of PC-Cl3 cells was inhibited. ER stress inducers also down-regulated thyroid-specific genes encoding thyroglobulin, thyroperoxidase, thyroid transcription factors TTF-1, TTF-2, and Pax-8 in these thyrocytes (53). This coincides with the establishment of a mesenchymal, stem-like phenotype consistent with EMT and included the down-regulation of E-cadherin transcripts, the up-regulation of mRNAs encoding vimentin, α-smooth muscle actin, the formation of actin stress fibers, and the loss of trans-epithelial resistance. All of these EMT events in thyroid cancer cells were stimulated with thapsigargin and tunicamycin (53). ER stressors likely facilitate EMT in different ways. In human kidney proximal tubular cells, this was shown to involve the induction of GRP78, GRP94, and phospho-eIF2α with subsequent EMT phenomenon (54). Thapsigargin and tunicamycin can also deregulate the intracellular Ca^2+^ metabolism, up-regulate the pleckstrin member T cell death-associated gene 51 (TDAG51), which sensitizes human kidney proximal tubular cells to mesenchymal transformation via Wnt signaling and primes these cells for TGF-β1-induced EMT response (54).

### Autophagy and EMT

Autophagy is considered a lysosomal degradation pathway responsible for the digestion of intracellular materials and the recycling of damaged cellular organelles (55, 56). Up to now, three types of autophagy have been described; these including macro-autophagy, micro-autophagy, and chaperone-mediated autophagy (CMA). They differ in their physiological functions and the pathways involved in their degradation mechanisms. Macro-autophagy includes a process of engulfing cellular waste in autophagosomes, which subsequently fuse with lysosomes and form autophagolysosomes, whereas micro-autophagy involves the direct uptake of cytoplasm by lysosomal membranes. CMA is considered the most selective form of autophagy and has so far only been described in mammalian cells. CMA involves a chaperone-guided process of internalization of soluble proteins by lysosomes [reviewed in Ref. (55, 57, 58)].

Physiologic basal autophagy plays an essential role in cellular homeostasis. It enables the cells to break down long-lived proteins and tightly regulates organelle turnover. Recycling of ER and mitochondria prevents ER stress and the accumulation of reactive oxygen and nitrogen species. During metabolic stress conditions, autophagy is initiated to sustain cellular energy in form of adenosine triphosphate (ATP) required for survival (59). Selective autophagy assists cells to rid themselves of damaged organelles, toxic protein aggregates (59), and invading microorganisms [reviewed in Ref. (60, 61)]. Autophagy is induced by an initial membrane nucleation that requires the ULK1 complex, and a class III phosphoinositide 3-kinase (PI3K) complex, which includes Bcl2 proteins member beclin 1 (62, 63). The isolation membrane selects its cargo and elongates until its edges fuse to form a double-membrane structure known as the autophagosome. Two ubiquitin-like conjugation systems, Atg5–Atg12 and microtubule-associated protein-1 light chain 3-phosphatidylethanolamine (LC3-II), are essential for the elongation of the isolation membrane to occur (51, 64–66). The autophagosome matures by fusing with endosomes and lysosomes to finally form the autophagolysosome where cargo degradation occurs (32, 67, 68).

Autophagy plays an essential function in tumor progression, metastasis, and the inhibition of cancer cell death (69, 70). While EMT promotes several mechanisms facilitating tumor invasiveness and increased cancer cell survival under stress conditions (71, 72), the association between EMT and autophagy in cancer invasiveness is less clear. Autophagy is involved in the regulation of epithelial plasticity (70). EMT is a form of enhanced epithelial plasticity and known to increase therapeutic resistance of cancer cells to cytotoxic agents and/or radiation. A strong link has recently been demonstrated between EMT, autophagy, stem-like characteristics, and resistance of cancer cells to cytotoxic T cell-induced killing, and targeting autophagy may help avoid immune resistance in breast cancer (73). Whether cancer (stem) cells opt for an aggressive phenotype, choose to enter an inactive state supported by autophagy, or endure cell death depends on the activation of different intracellular pathways and specific changes in gene expression profiles upon external stimuli (74). The hedgehog signaling pathway is one example of a cellular signaling system balancing invasion versus autophagy. Active hedgehog signaling promotes an aggressive phenotype (75), while its inhibition activates autophagy (76). Another example is the activation of 5′ adenosine monophosphate-activated protein kinase (AMPK) which reduces tumor cell invasion (77) and induces autophagy in response to genotoxic stress (78) and nutrient starvation (79). In hepatocarcinoma cells, the induction of autophagy represses the expression of epithelial markers but promotes the expression of mesenchymal markers and the activation of the TGF-β/Smad3-dependent signaling pathway (80). Importantly, data from numerous cell systems, including thyroid cancer, identify autophagy as an important new player in EMT plasticity, therapeutic resistance, and metastasis. Clearly, the role of autophagy and ER stress/UPR responses in thyroid (cancer) stemness is a newly emerging and exiting field with potentially important innovation in the treatment of thyroid cancer.

### Damage-associated pattern recognition in cancer stem cells

Damage-associated molecular patterns are a group of endogenous molecules acting as danger signals to initiate cellular repair and survival mechanisms (81, 82). Endogenous soluble molecules released during cell damage, necrosis, and cell stress are referred to as alarmins (82, 83). DAMP signaling induces an early immune response involving the innate and adaptive immune system, thus, initiating a “sterile inflammatory” reaction (83). In contrast, pathogen-associated molecular patterns (PAMPs) are exogenous danger signals derived from pathogens which activate diverse cellular receptors of the innate immune system to initiate host defense mechanisms (83). DAMPs may be exposed at the cell surface, e.g., calreticulin (CRT) and heat shock protein 90 (HSP90), or are secreted from cells, e.g., ATP, S100A8/A9 proteins, and high-mobility group box 1 protein (HMGB1). Secreted by tumor cells, these factors promote tumor growth and chemoresistance (81, 84–86); for a comprehensive list of DAMPs and their receptors, see Ref. (85).

Damage-associated molecular patterns act through membrane-anchored pattern recognition receptors (PRR) which recognize a structural pattern rather than a specific ligand. Examples for PRR are the NOD-like receptors (NLRs), toll-like receptors (TLRs), and the receptor for advanced glycation end products (RAGEs) (21, 87, 88). Several PRR, in particular TLRs and RAGE, are expressed in cancer cells and share distinct DAMP ligands such as HMGB1 and S100 proteins (89). In leukocytes or dendritic cells, PRR signaling results in the release of cytokines (innate immunity) or the initiation of adaptive immune responses. Interestingly, several PRR such as the TLRs recognize both PAMP and DAMP ligands (83). Also, DAMPs can interact with PAMPs to activate several PRR (90), suggesting the utilization of common cellular pathways when sensing exogenous and endogenous dangers.

All TLRs comprise a leucine-rich repeat extracellular domain responsible for ligand binding and a transmembrane domain and an intracellular toll/IL-1 receptor (TIR) domain for signal transduction. Ligand-binding results in the homo- or hetero-dimerization of TLRs as a requirement for intracellular signaling (87). Engaging with the intracellular TIR domains of dimerized TLRs are five cytoplasmic adaptor molecules which activate intracellular signaling, including NF-κB and MAPK pathways. These intracellular TLR signaling adaptors are myeloid differentiation factor 88 (MyD88), MyD88 adapter-like protein (MAL), TIR domain-containing adaptor protein inducing interferon-beta (TRIF), TRIF-related adaptor molecule (TRAM), and sterile α- and armadillo-motif-containing protein (SARM) [reviewed in Ref. (82, 91)]. MyD88 is used by all TLRs and was shown to have a particular role in tumorigenesis (92).

Receptor for advanced glycation end product belongs to the immunoglobulin-like family of transmembrane receptors (88, 93) and is expressed in several human cancers (94). The three extracellular domains of RAGE, the V-, C1-, and C2-domain, function in ligand binding (93). The single transmembrane domain connects to the short cytoplasmic tail of RAGE which interacts with the cytoplasmic adaptor molecule diaphanous-1 (Dia-1) (95) and with toll-interleukin 1 receptor domain-containing adaptor protein (TIRAP) and MyD88; the latter two are also adapters for TLR 2 and 4 (96). Depending on the ligand and cell context, activated RAGE can signal through multiple signaling pathways, including extracellular ERK1/2, p38 MAP kinase, Cdc42/Rac-1, c-Jun-NH2-terminal kinase/stress-activated protein kinase (JNK/SAPK). This finally results in the activation of NF-κB and the induction of pro-inflammatory and pro-migratory responses (97). RAGE ligands include HMGB1, S100/calgranulin proteins, advanced glycation end products (AGEs), and amyloid β proteins (98). RAGE signaling can be modified by soluble RAGE species. Endogenously produced soluble RAGE splice variant (esRAGE), unable to insert into the membrane, is secreted from cells and acts as a decoy for RAGE ligands (88). Cleavage of the extracellular domains by a disintegrin and metalloproteinase 10 (ADAM10) releases soluble RAGE which can also act as scavenger for ligands (99). Unfortunately, soluble RAGE species are not reliable clinical predictors of outcome in cancer or inflammatory diseases (88). Ligand binding to RAGE induces RAGE expression in a positive feedback loop and promotes clustering of RAGE at the cell surface leading to sustained RAGE signaling by multimeric ligands (97, 100, 101). In the thyroid gland, RAGE is not expressed in normal follicular epithelial cells, but its expression is up-regulated in thyroid epithelial cells of follicular adenoma and thyroid carcinoma (102) suggesting that hyperactive and neoplastic thyroid cells respond to DAMPs.

Utilizing HMGB1 as an example, we demonstrate how DAMP–PRR signaling can influence tumorigenesis and stem cell functions. HMGB1 is a non-histone DNA-binding protein of the high-mobility group protein family and is composed of two DNA-binding HMG-box motives and an acidic C-terminus. Originally described as an exclusively nuclear protein involved in the modulation of gene transcription and chromosomal stability, HMGB1 is now known to have a cytoplasmic role in regulating autophagy, cell survival, and EMT (103). HMGB1 is passively released from dead or injured cells and actively secreted from immune cells and cancer cells in response to cellular stress signals (83, 103). HMGB1 secretion is modulated by various post-translational modifications and by secondary messengers such as cytosolic calcium, nitric oxide, and reactive oxygen species (ROS) (103). Extracellular HMGB1 binds to several receptors, including TLR2, TLR4, and RAGE (89), to promote cell migration, proliferation, and differentiation. Secreted HMGB1 functions in the repair of tissue damage and chronic inflammation and leads to increased tumor growth and metastasis, promotes angiogenesis, regulates vascular remodeling, and enhances stem cell renewal (88, 89, 104). The influence of HMGB1 on stem/progenitor cells is mediated via RAGE and TLRs and results in enhanced stem cell functions (104–106).

HMGB1–RAGE–NF-κB signaling promotes neuronal stem cell proliferation/differentiation (107) and enhances xenograft metastasis via EMT and the activation of MAPK pathways (108). Astrocyte-derived HMGB1 enhances stem cell recruitment in the brain during stroke recovery (109, 110). HMGB1–TLR2 signaling via signal transducer and activator of transcription factor 3 (STAT3) and Smad3 activation enhances breast cancer stem cell self-renewal and increases breast cancer metastasis (104). HMGB1 aids in the recruitment of endothelial precursor cells (EPC) using RAGE (111) and TLR2/TLR4 in c-kit-positive EPC (112), thus, contributing to tissue repair and tumor growth. In PTC, the HMGB1–RAGE interaction increases the expression of the microRNAs miR221 and miR222 and promotes PTC cell growth and motility by inhibiting the cell cycle regulator p27kip1 (113).

The role of TLR in cancer is not fully understood. TLR receptor activation by PAMPs and distinct endogenous (DAMPs) signals has been associated with either tumor promoting or anti-tumor activities in human cancers (114, 115). Very little information exists on the function of DAMP–PRR interactions in the thyroid gland and in thyroid cancer (stem) cells. Normal human and rat thyroid cells express TLR 2 and TR4. Activation of TLR in the rat thyroid cell line FRTL-5 induces NF-κB activation and secretion of the pro-inflammatory cytokines interferon-beta (IFN-β), indicating that the thyroid gland is capable of responding to DAMPs and PAMPs with the initiation of “sterile inflammatory responses” to promote proliferation and angiogenesis in the thyroid (116). In FTC, TLR4 was localized to inflammatory tissue regions surrounding the tumor and TLR4 expression was associated with metastasis in FTC patients (117). TLR10 polymorphism has been associated with increased tumor size in patients with PTC (118). In mouse breast progenitor cells, PAMP ligands of TLR4 enhance cell proliferation and mammosphere growth (119), whereas similar activity in intestinal stem cells induces a p53-dependent apoptosis (120). Also, flagellin–TLR5 signaling in human breast cancer cells reduces cell proliferation and anchorage-independent growth in human breast cancer xenografts (121). TLR4 activation was shown to suppress TGF-beta signaling and promote chemoresistance in tumor-initiating cells of virus-induced hepatocellular carcinoma (21). Cancer stem cells may respond to DAMPs differently from normal stem cells and the nature of the ligand affects the cellular responses to PRR activation.

In summary, cross talk between different PRR, the activation of several PRR by the same DAMP, and the response of one PRR to more than one DAMP altogether suggest that cellular responses to DAMPs are: (i) (stem) cell-type and tissue-specific, (ii) influenced by the nature of the cellular stresses, and (iii) affected by the spatial and temporal distribution and concentration of DAMPs. It is intriguing to speculate that the complex DAMP–PRR system with its functional roles in cell plasticity, autophagy, and cellular stress control represents an important and as yet under-valued novel topic in stem cell research and a potentially lucrative therapeutic target to manipulate cancer stem cell functions.

## High-Mobility Group A2: A Link Between Stemness, EMT, and Chemoresistance

The low molecular weight HMGA2, formerly named HMGI-C, is a member of the HMGA family of non-histone nuclear protein, which also includes HMGA1 and its splice forms. Structural analysis of HMGA2 revealed three DNA-binding domains, called AT-hook motifs, which facilitate binding to AT-rich regions of the minor groove of B-form DNA (122, 123). Blockage of HMGA2 can prevent the transformation of rat thyroid cells by murine retroviruses (124). HMGA2 is a marker of stem cells, absent in most adult tissues, and re-expressed in many cancer (stem) cells (125–129). HMGA2 binds to the AT-rich G-bands in the chromosomes and to centromeres and telomeres of metaphase preparations (130). Chromosomal rearrangements of both HMGA1 and HMGA2 proteins have been correlated with neoplastic transformation. HMGA2 overexpression, dysregulation, or truncation has been linked to benign and malignant tumors. This includes benign mesenchymal tumors (131), uterine leiomyomata (127, 132), pituitary adenoma (132), human prolactinoma (133), pancreatic cancer (134, 135), retinoblastoma (136), embryonic rhabdomyosarcoma (80), chondrosarcoma (137), lung cancer (138), and hepatocellular carcinoma (104). In thyroid cancer, elevated levels of HMGA2 are considered a molecular marker to distinguish benign and malignant thyroid neoplasms (139, 140).

### Protective roles of HMGA2

Multifunctional HMGA1 and 2 are localized in the nucleus and regulate transcriptional genes activity, DNA replication, and DNA repair (141). HMGA2 is a new member of the BER protein complex and interacts with human AP endonuclease 1 (APE1) to promote chemoresistance in cancer cells, including human undifferentiated thyroid cancer cells (16). HMGA1 and 2 serve as substrate for the phosphatidylinositol 3-kinase-related kinase (PIKK) family member ataxia telangiectasia mutated (ATM) and downstream checkpoint kinase 2 (CHK2) which are important for DNA damage signaling. Upon exposure to genotoxicants, increased HMGA1/2 expression correlates with increased ATM expression levels and enhanced cellular DNA damage response (142, 143). HMGA2 also interacts with ataxia telangiectasia and Rad3-related kinase (ATR) and HMGA2-mediated activation of the ATR–checkpoint kinase 1 (CHK1) signaling pathway with resulting G2/M arrest increases chemoresistance against BER-inducing genotoxic alkylating agents in human thyroid and other cancer cells (17). Recently, we showed that HMGA2 plays an important novel role in protecting the integrity and functionality of arrested replication forks in cancer cells. HMGA2 preferentially binds with higher affinity to DNA Y- (replication fork) and X- (Holliday junctions) structures typically observed at replication forks (45). Binding of HMGA2 to these DNA conformations protected stalled replication forks from endonuclease digestion and conferred a survival advantage onto HMGA2^+^ cancer cells, including thyroid cancer cells, when exposed to chemotherapeutics such as hydroxyurea used in the treatment of cancer patients (45).

### HMGA2 in stemness and EMT

Cancer initiating cells (CIC) evade cell death and attenuate the cytotoxic effects of radio- and chemotherapy by modulating DNA damage repair mechanisms to promote therapeutic resistance and tumor recurrence (80, 144). The Lin28–HMGA2–let-7 axis plays a significant role in promoting EMT, tissue invasion/metastasis, and therapeutic resistance in cancer cells (21). Ectopically expressed of the microRNA binding protein, Lin28 down-regulates the miR let-7 which attenuates the inhibitory let-7-mediated association with the 3′ untranslated region of HMGA2 mRNA, resulting in the up-regulation of HMGA2 protein (145). Presence of HMGA2 in retinoblastoma and pancreatic cancer cells was shown to enhance cell proliferation and increase stemness (135, 136). HMGA2 also has a key role in maintaining high self-renewing capacity in hematopoietic stem cells and this involves the inhibition of the micro RNA let-7 by Lin28 (146). Let-7 suppresses both H-Ras and HMGA2 and this causes reduced proliferation and differentiation of breast CIC (44). In addition to the phenotypic and/or cell surface markers like CD133, CD34, CD90, SOX2, OCT4, and NANOG, functional markers such as the aldehyde dehydrogenase (ALDH) activity also determine the stemness in a cancer sub-population. The inhibition of ALDH activity reduces stemness and sensitizes CIC to cytotoxic insult (80). Cellular levels of HMGA2 directly correlate with ALDH activity in breast cancer and contribute to tumor migration and resistance toward DNA-damaging agents (21). HMGA2 is found at the invasive front of the tumor to promote tissue invasion and tumor recurrence following therapy (147). HMGA2-induced EMT is promoted by TGF-β signaling (147) and the pro-inflammatory cytokine oncostatin M (OSM) (148) which signal via the STAT3 (148), the Ras/MAPK signaling pathway (135), and Twist and Snail, the two key regulators of EMT and major contributors to metastasis and tumor recurrence (104, 149). Suppression of Lin28 or HMGA2 increases let-7 and this reverses EMT and affects the level of therapeutic resistance. HMGA2^+^ cancer cells were shown to exhibit resistance against 4 of 11 anti-cancer drugs tested (128).

In conclusion, cancer (stem) cells capable of utilizing HMG proteins to enhance DNA repair mechanisms in response to DNA-damaging drugs are likely contributing to therapeutic resistance to anti-cancer treatments in metastatic or recurrent cancers. Modulation of the EMT, ER stress, and autophagy regulation in cancer (stem) cells serves as important survival strategy in avoiding apoptosis under nutritional or oxygen deprivation. Similarly, DAMP signaling via several PRR may serve as another tumor survival response by controlling cell proliferation, inflammatory, and autophagy responses in cancer (stem) cells.

## Conflict of Interest Statement

The authors declare that the research was conducted in the absence of any commercial or financial relationships that could be construed as a potential conflict of interest.
